# The prognostic value of *BRCA1* promoter methylation in early stage triple negative breast cancer

**DOI:** 10.7243/2049-7962-3-2

**Published:** 2014-03-19

**Authors:** Priyanka Sharma, Shane R. Stecklein, Bruce F. Kimler, Geetika Sethi, Brian K. Petroff, Teresa A. Phillips, Ossama W. Tawfik, Andrew K. Godwin, Roy A. Jensen

**Affiliations:** 1 Division of Hematology/Oncology, Department of Internal Medicine, University of Kansas Medical Center, Kansas City, Kansas, USA; 2 Department of Pathology and Laboratory Medicine, University of Kansas Medical Center, Kansas City, Kansas, USA; 3 Department of Radiation Oncology, University of Kansas Medical Center, Kansas City, Kansas, USA; 4 Breast Cancer Prevention Center, University of Kansas Medical Center, Kansas City, Kansas, USA; 5 The University of Kansas Cancer Center, University of Kansas Medical Center, Kansas City, Kansas, USA; 6 Department of Biochemistry and Molecular Biology, Drexel University College of Medicine, Philadelphia, Pennsylvania, USA

**Keywords:** Triple negative breast cancer, *BRCA1* promoter methylation, prognosis, chemosensitivity, biomarker

## Abstract

**Introduction:**

Methylation of the *BRCA1* promoter is frequent in triple negative breast cancers (TNBC) and results in a tumor phenotype similar to *BRCA1*-mutated tumors. *BRCA1* mutation-associated cancers are more sensitive to DNA damaging agents as compared to conventional chemotherapy agents. It is not known if there is an interaction between the presence of *BRCA1* promoter methylation (PM) and response to chemotherapy agents in sporadic TNBC. We sought to investigate the prognostic significance of *BRCA1* PM in TNBC patients receiving standard chemotherapy.

**Methods:**

Subjects with stage I-III TNBC treated with chemotherapy were identified and their formalin-fixed paraffin-embedded (FFPE) tumor specimens retrieved. Genomic DNA was isolated and subjected to methylation-specific PCR (MSPCR).

**Results:**

DNA was isolated from primary tumor of 39 subjects. *BRCA1* PM was detected in 30% of patients. Presence of *BRCA1* PM was associated with lower *BRCA1* transcript levels, suggesting epigenetic *BRCA1* silencing. All patients received chemotherapy (anthracycline:90%, taxane:69%). At a median follow-up of 64 months, 46% of patients have recurred and 36% have died. On univariate analysis, African-American race, node positivity, stage, and *BRCA1* PM were associated with worse RFS and OS. Five year OS was 36% for patients with *BRCA1* PM vs. 77% for patients without *BRCA1* PM (p=0.004). On multivariable analysis, *BRCA1* PM was associated with significantly worse RFS and OS.

**Conclusions:**

We show that *BRCA1* PM is common in TNBC and has the potential to identify a significant fraction of TNBC patients who have suboptimal outcomes with standard chemotherapy.

## Introduction

Triple negative breast cancer (TNBC) is defined by the lack of expression of estrogen receptor (ER) and progesterone receptor (PR), and absence of *ERBB2* (HER2) over expression and/or gene amplification and is associated with poor long-term outcomes compared to other breast cancer subtypes [1-3]. Despite receiving standard cytotoxic chemotherapy, a significant proportion (approximately 30-40%) of patients with early stage TNBC develop metastatic disease and succumb to their cancer [4-6]. To improve outcomes for this subtype, we not only need novel targeted agents, but also need to identify predictors of response/resistance to standard chemotherapy. *BRCA1* dysfunction may have the potential to serve both as a therapeutic target and as prognostic marker of response to targeted therapy in TNBC.

*BRCA1* is a classic tumor suppressor gene and the loss of the wild-type allele [loss of heterozygosity (LOH)] is required for tumorigenesis in germline mutation carriers. Sporadic TNBC and *BRCA1* germline mutation-associated breast cancers share many histopathologic and molecular features; however, only 10-20% of TNBCs harbor germline *BRCA1* mutation [7-9]. The phenotypic and molecular similarities between *BRCA1* mutation-associated and sporadic TNBC have led many to surmise that sporadic TNBCs may involve *BRCA1* pathway dysfunction through non-mutational means. Epigenetic inactivation of tumor suppressor genes by the aberrant addition of methyl groups in their CpG-rich regulatory regions (promoter CpG islands) is a common hallmark of human tumors. Hypermethylation of the *BRCA1* promoter has been proposed as one of the mechanisms for functionally inactivating the *BRCA1* gene in breast cancers and this epigenetic inactivation of *BRCA1* is associated with a gene expression profile similar to that of inherited *BRCA1* mutation-associated breast cancer [10-12].

*BRCA1* promoter methylation (PM) is observed in 20-60% of sporadic TNBC and may be an important mechanism contributing to the loss of *BRCA1* function in sporadic TNBC [11,13-15]. Methylation specific PCR (MSPCR) has been utilized to detect hypermethylation of the areas of interest in the CpG islands of the *BRCA1* promoter by many investigators [10,11,14]. MSPCR is relatively inexpensive and can be performed on genomic DNA derived from formalin-fixed paraffin-embedded (FFPE) tissue, and thus has the potential of being easily applied to clinical settings.

*BRCA1* plays a crucial role in homologous recombination-dependent DNA double-strand break and interstrand crosslink repair, and *BRCA1*-deficient cells are particularly susceptible to the DNA damaging agents like platinum compounds [16,17]. Observational studies and small neoadjuvant studies have also suggested that *BRCA1* mutation-associated breast cancers may be more sensitive to platinum agents as compared to sporadic TNBC [8,18]. It is not known if epigenetic silencing of *BRCA1* via promoter methylation in sporadic TNBC impacts response to chemotherapy. Several prior studies have evaluated *BRCA1* PM in TNBC but, have shown conflicting results in regards to prognostic impact of *BRCA1* PM in TNBC [15,19,20-22]. These prior studies, differ in the methodology used for detection of *BRCA1 PM*, do not uniformly include analysis of *BRCA1* expression (to confirm epigenetic gene silencing) and include TNBC patients treated with various different chemotherapy regimens thus, limiting the ability of cross study comparisons. The purpose of this study was to investigate the prognostic significance of epigenetic *BRCA1* silencing in early stage TNBC patients treated with modern chemotherapy (anthracyline and taxane).

## Methods

### Ethics statement

This study was approved by the Institutional Review Board (IRB) at the University of Kansas Medical Center, Kansas City, Kansas, USA, and was exempt from the informed consent process pursuant to 45 CFR 46.11(d).

### Patients

Subjects with early stage (TNM stage I-III) TNBC who had definitive surgery at the University of Kansas Hospital, were treated with adjuvant/neoadjuvant chemotherapy, and for whom tumor specimens were available in our pathology archives were identified. TNBC was defined as negative ER, PR, and HER2 status. Immunohistochemical nuclear staining of less than or equal to 1% was considered a negative result for ER and PR (in accordance with 2010 ASCO/CAP guidelines). HER2-negative tumors were defined as 0 or 1+ on IHC staining and/or lack of gene amplification found on FISH testing (ratio less than 2.0).

**Figure 1:**
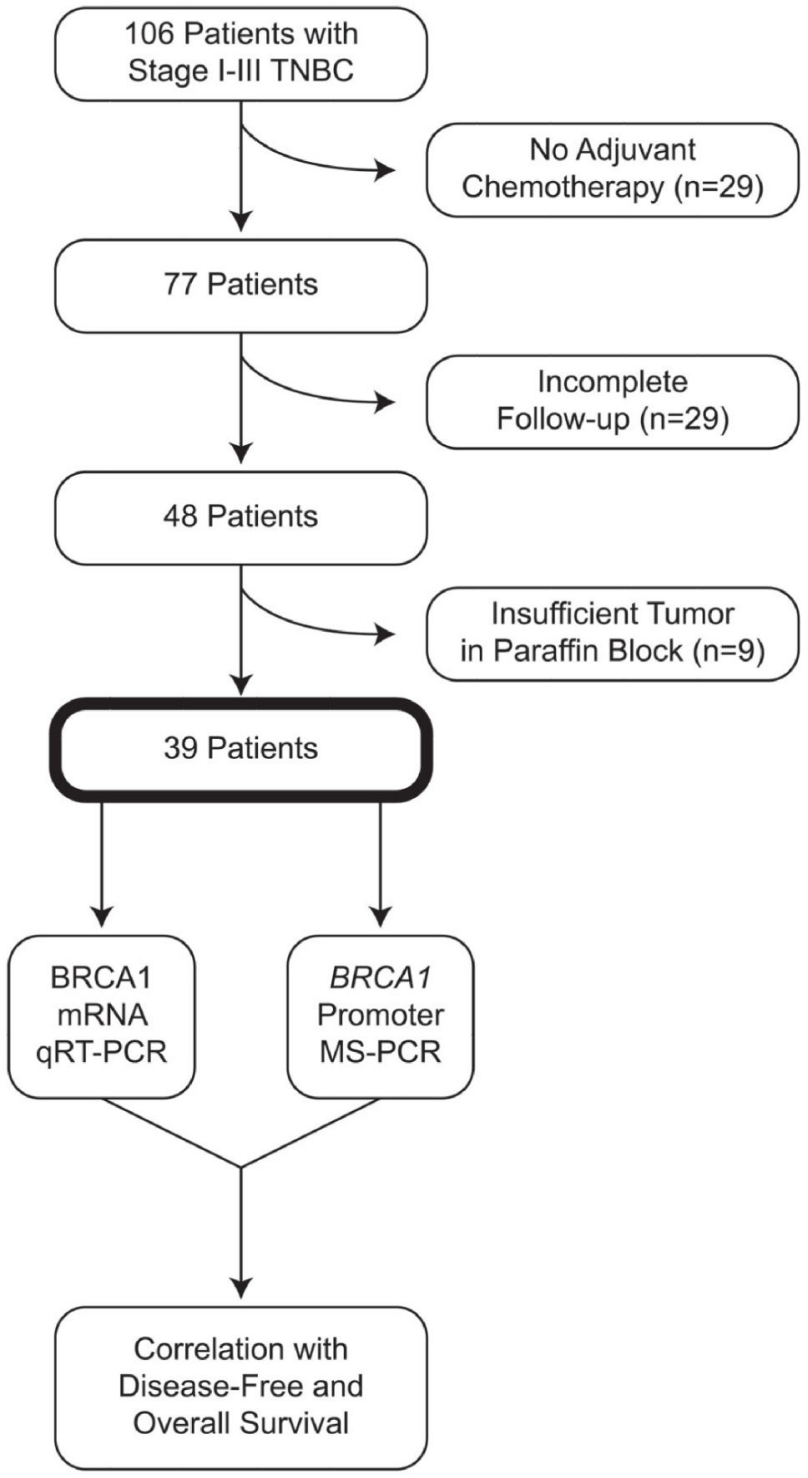
Identification of tumor specimens for analysis.

Under an IRB-approved protocol, 106 patients with stage I-III TNBC who had definitive surgery at our institution between 1996-2008 were identified. 29/106 patients did not receive any systemic adjuvant/neoadjuvant chemotherapy and another 29 patients had incomplete information on follow up. FFPE tumor tissue samples were retrieved from the pathology archives for the remaining 48 patients. Each tumor specimen was evaluated by a pathologist to confirm the presence of invasive disease and only samples with >50% invasive cancer were included in the analysis. Thirty-nine of the 48 patients had an archived tissue block available with adequate invasive cancer and formed the study cohort (Figure 1). For patients who received neoadjuvant chemotherapy, the biopsy specimen obtained prior to initiation of neoadjuvant chemotherapy was utilized for evaluation.

Demographic and clinical information regarding patho-logical stage, breast cancer treatment, outcome etc. was collected by review of the medical charts.

### *BRCA1* promoter methylation (*BRCA1* PM)

**Figure 2:**
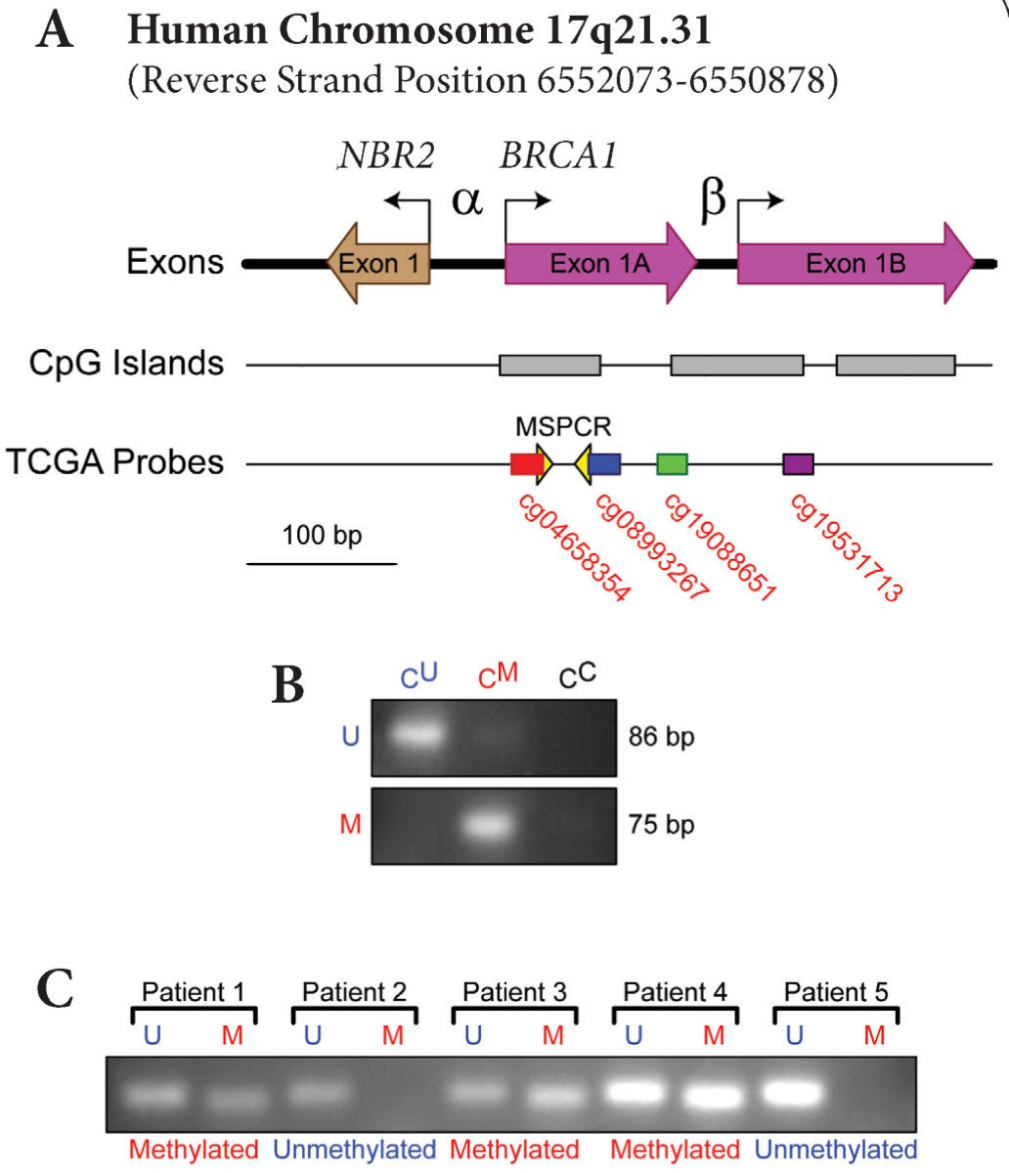
(**A**) Diagram of *BRCA1* promoter locus and region interrogated by MSPCR assay. NBR2 is the “neighbor of *BRCA1* gene 2” ORF. α and β are the two promoters of the human *BRCA1* gene and α bidirectonally regulates NBR2. CpG islands were predicted using MethPrimer (Li Laboratory, UCSF) using observed/expected ratio >0.6 and %GC >50. The region amplified in our assay is denoted under “MSPCR”. Illumina Human Methylation27 probes located within the promoter region of *BRCA1* are noted by probe identification number. (**B**) Specificity controls for the MSPCR reaction. Unconverted genomic DNA (C^C^), universally unmethylated bisulfite-converted genomic DNA (C^U^) and universally methylated bisulfite-converted (C^M^) were amplified with primers specific for bisulfite-converted unmethylated (U) or methylated (M) *BRCA1* promoter. (**C**) *BRCA1* promoter MSPCR electrophoresis images from five representative patient samples. “U” and “M” indicate reactions with unmethylated-specific and methylated-specific primers, respectively.

Tumor-dense areas of 20 μm FFPE tissue sections were manually dissected and genomic DNA (gDNA) was isolated and bisulfite converted using the EpiTect® Plus FFPE Bisulfite Kit (Qiagen). Purified converted DNA was subjected to methylation-specific PCR (MSPCR) using the EpiTect® MSP Kit (Qiagen). The unmethylated template primers were (forward) TTGGTTTTTGTGGTAATGGAAAAGTGT and (reverse) CAAAAAATCTCAACAAACTCACACCA, resulting in an 86 base pair PCR product. The methylated template primers were (forward) TCGTGGTAACGGAAAAGCGC and (reverse) AAATCTCAACGAACTCACGCCG, resulting in a 75 base pair PCR product. These primers have been extensively characterized by previous groups (Figure 2A) [10,23]. PCR conditions were as follows: 95.0°C for 10 minutes, then 35 cycles of 94.0°C for 15 seconds, 55.0°C for 30 seconds, 72.0°C for 30 seconds, and a final extension at 72.0°C for 10 minutes. PCR products were electrophoresed on a 2.5% agarose gel stained with ethidium bromide and visualized on a UVP Bioimaging system. Specificity of the reactions was confirmed using the EpiTect® Control DNA set (Qiagen) with the same primers and PCR conditions. The presence of a methylated band was recorded as “positive” for *BRCA1* PM (Figures 2B-2C).

### *BRCA1* mRNA quantitative real-time PCR (qRT-PCR)

Total RNA was isolated using the RecoverAll™ kit (Life Technologies), which includes DNAse treatment performed to remove genomic DNA. RNA was reverse transcribed to cDNA using SMARTScribe™ reverse transcriptase (Clontech) and random nonamer primer. cDNAs were assayed in duplicate for expression of *BRCA1* transcript levels as well as reference transcripts using specific primer and probe sets (TaqMan® Gene Expression Assays; Life Technologies) and TaqMan® chemistry [24]. Cycle threshold (Ct) values were calculated for each endpoint, corrected for housekeeping gene expression (cyclophillin A and hypoxanthine phosphoribosyltransferase 1) and relative gene expression was calculated using the ΔΔCt method. Expression is reported as multiples of the median.

### Analysis of TCGA dataset

BRCA1 gene expression (Agilent platform) and DNA methylation (Human Methylation27 and Human Methylation450 arrays) data for TNBC breast cancer specimens were obtained from the TCGA database and analyzed as described previously [25]. Briefly, the z-scores for BRCA1 mRNA expression and beta values for DNA methylation (four probes spanning the promoter region of interest: cg04658354, cg08993267, cg19088651 and cg19531713) for 56 TNBC samples (for which both expression and methylation data were available at the time of analysis) were downloaded from TCGA portal (Figure 2A) (http://tcga-data.nci.nih.gov/tcga/tcgaHome2.jsp). Correlation analysis between BRCA1 mRNA expression and *BRCA1* promoter DNA methylation (at each of the four CpG islands individually and the mean beta value of all four probes) was performed using GraphPad Prism.

### Statistical analysis

Patient characteristics were compared between groups (*BRCA1* PM present vs. *BRCA1* PM absent) by a chi-square test or Wilcoxon’s rank-sum test, as appropriate. Time to recurrence was measured from the date of diagnosis to the date of local or systemic recurrence or the last follow-up. Overall survival (OS) time was measured from the date of diagnosis to the date of death, or the last follow-up.

Survival outcomes were estimated according to the Kaplan–Meier method and compared between groups by the log-rank statistic. Cox proportional hazards models were fit to determine the association of *BRCA1* PM with the risk of recurrence and death after adjustment for other characteristics.

## Results

### Study population

Under an IRB-approved protocol, 48 patients with stage I-III TNBC who had definitive surgery at our institution between 1996-2008, were treated with adjuvant/neoadjuvant chemotherapy, and for whom tumor specimens were available in our pathology archives were identified. Thirty-nine of 48 subjects with TNBC had adequate tumor specimen available for analysis. Table 1 describes the baseline demographics of the study population. All patients received systemic chemotherapy for early stage disease (74% received adjuvant and 26% received neoadjuvant chemotherapy). For patients who received neoadjuvant chemotherapy, the biopsy specimen obtained prior to initiation of neoadjuvant chemotherapy was utilized for the study. Ninety percent (35/39) received an anthracycline and 69% (27/39) received a taxane as part of systemic therapy. All patients received adjuvant radiotherapy based on standard clinical guidelines.

### *BRCA1* promoter methylation (*BRCA1* PM) and expression analysis

**Figure 3:**
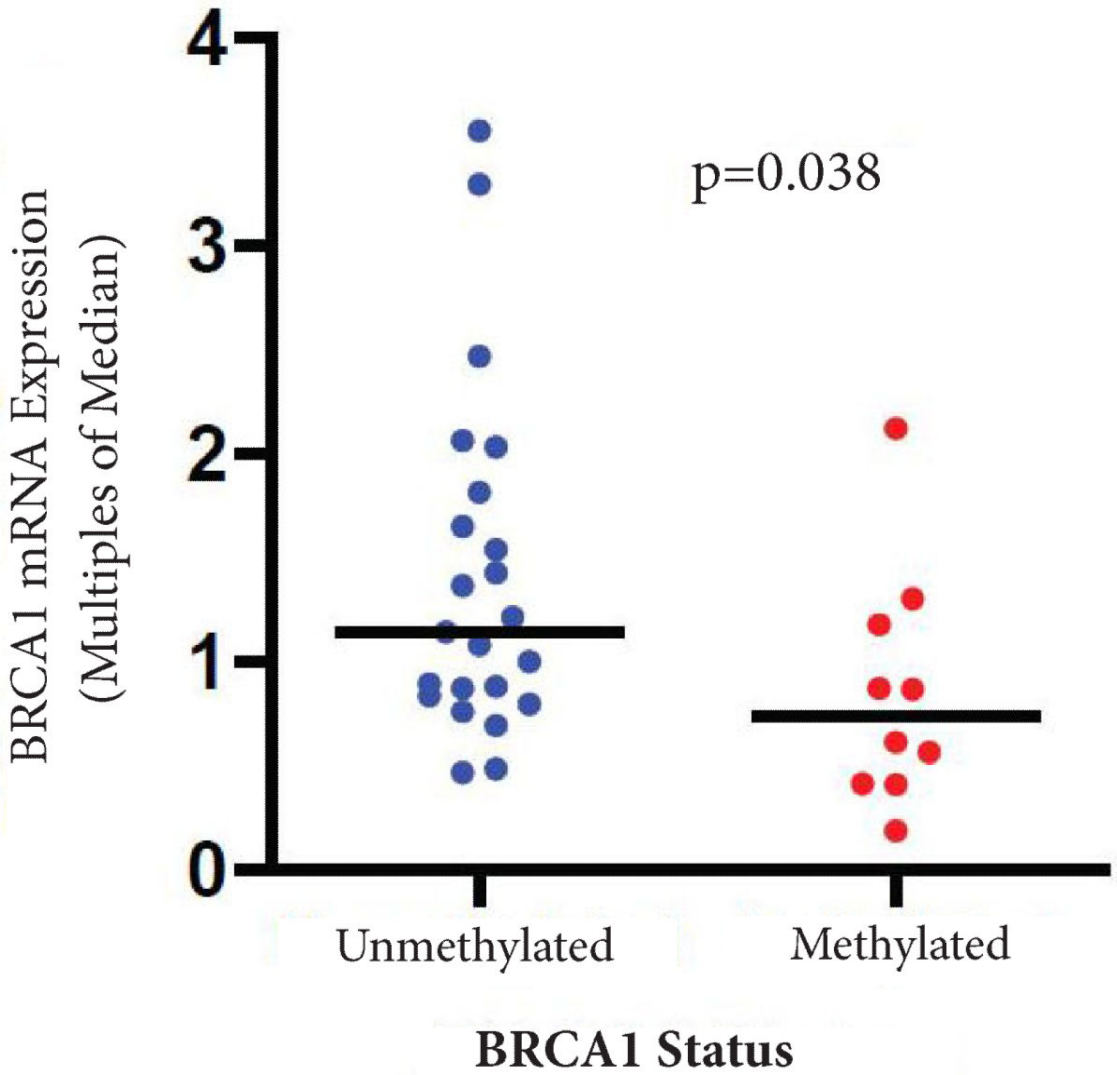
Association between *BRCA1* promoter methylation and *BRCA1* transcript levels. Lines note median expression value, p value represents Mann-Whitney test.

*BRCA1* PM MSPCR assay was successful in 95% (37/39) of specimens and BRCA1 mRNA qRT-PCR was successful in 92% (36/39) of specimens. *BRCA1* PM was detected in 30% (11/37) of subjects. There was no statistically significant association between presence of *BRCA1* PM and age, race, nodal status, lymphovascular invasion and clinical stage (Table 1). For 34 subjects with both *BRCA1* promoter methylation and *BRCA1* qRT-PCR data, the presence of *BRCA1* PM was associated with lower *BRCA1* transcript levels suggesting epigenetic silencing of *BRCA1* gene (median *BRCA1* expression was 0.74 multiples of the median in tumors with *BRCA1* PM compared to 1.14 in tumors without *BRCA*1 PM, p=0.038, Figure 3).

### Analysis of TCGA dataset for *BRCA1* PM and expression

**Figure 4:**
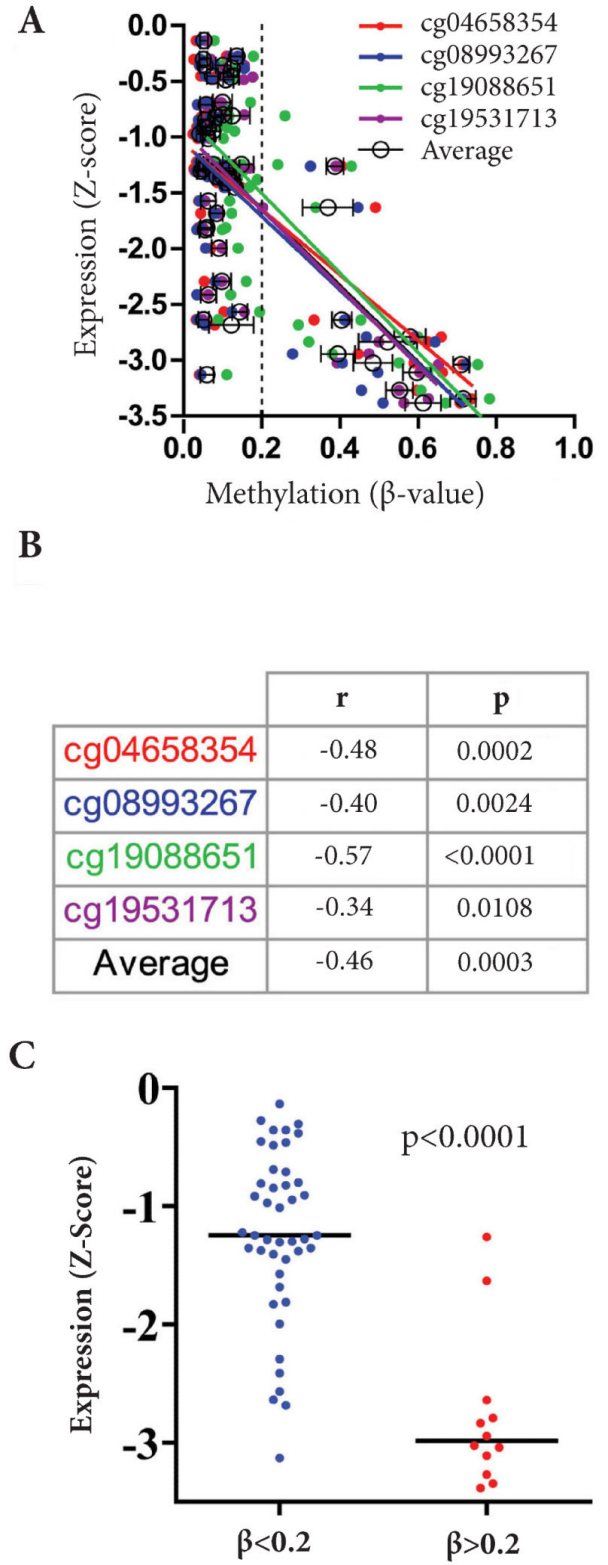
(**A**) Correlation analysis between *BRCA1* expression and methylation across 56 TNBC specimens (for which both expression and methylation data were available) obtained from the TCGA breast cancer database. (**B**) Correlation coefficients and significance of methylation and mRNA expression from TCGA dataset. (**C**) Association between *BRCA1* promoter methylation (determined by average TCGA probe β-value >0.2) and *BRCA1* mRNA expression. Lines note median expression value, p value represents Mann-Whitney test.

We analyzed the TCGA breast cancer dataset for 56 TNBC specimens for which both expression (z-score) and methylation (β-value) data were available. Among the four probes included in the TCGA dataset, two probes (cg04658354 and cg08993267) flank and overlap with the region queried by the MSPCR we used to interrogate *BRCA1* PM in our study. The two additional probes (cg19088651 and cg19531713) lay 105 and 307 base pairs downstream from the MSPCR locus, respectively (Figure 2A). There was a significant inverse correlation between methylation and *BRCA1* mRNA expression at all four probe sites individually, and when all were considered as a composite measure of methylation (Figures 4A-4B). There appeared to be a threshold at a composite β-value of approximately 0.2 (dotted line in Figure 4A), with expression of *BRCA1* being significantly lower (p<0.0001) in the 21% (12/56) of tumors with a methylation value beyond this threshold (Figure 4C). Taken together, these data confirm that hypermethylation in this region is strongly associated with epigenetic silencing of the *BRCA1* gene.

### *BRCA1* PM and outcome

**Figure 5:**
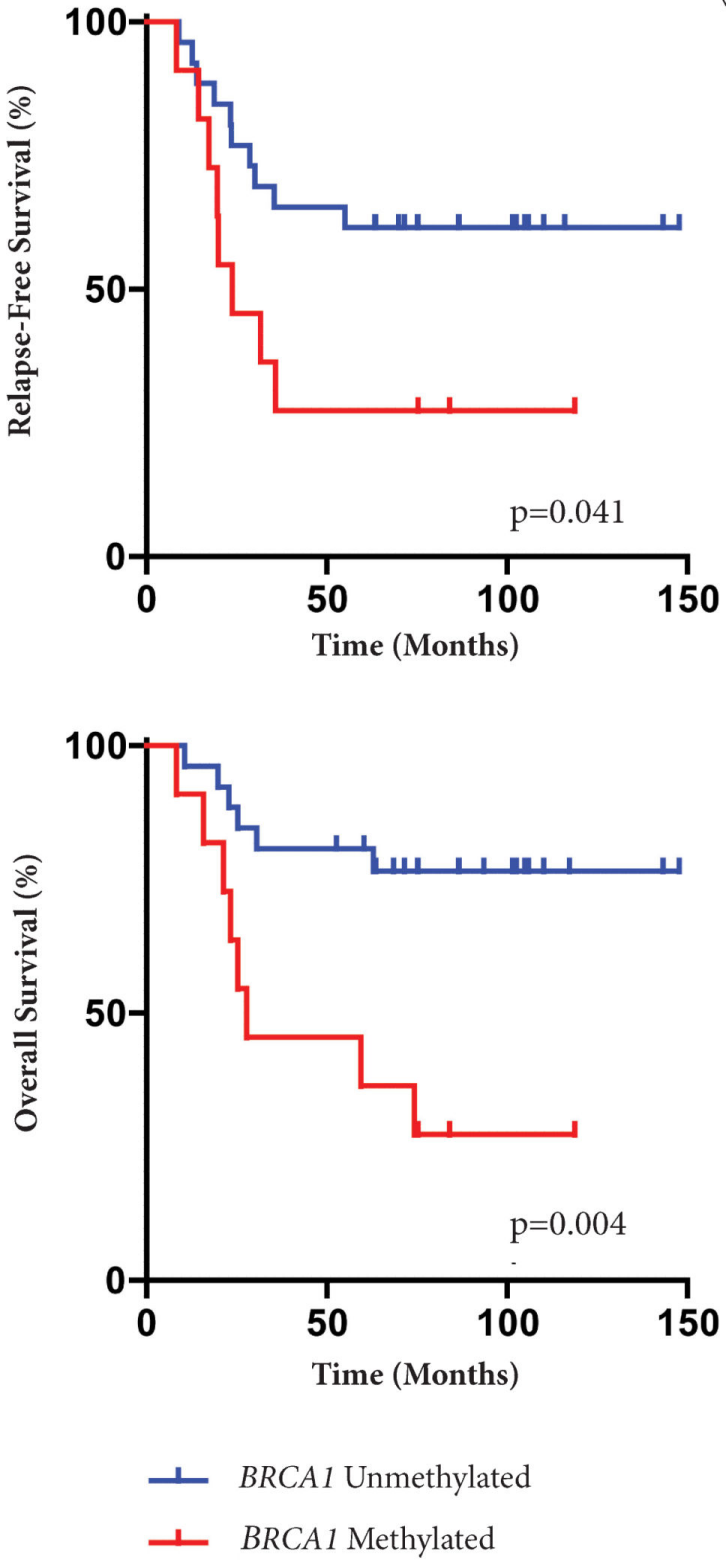
Kaplan-Meier survival plots for recurrence-free survival (RFS) and overall survival (OS) by methylation status. Tick marks denote time of censoring.

At a median and mean follow-up of 64 months (range 8-148 months) and 63 months, respectively, there have been 18 (46%) recurrences and 14 (36%) deaths. Survival estimates are summarized in Table 2. Node positivity, higher stage, African-American race and presence of *BRCA1* PM were associated with worse RFS and OS (univariate analysis). Chemotherapy regimens (taxane-containing vs. non-taxane-containing regimens and anthracycline-containing vs. non-anthracycline-containing regimens) did not impact RFS or OS (although this analysis is limited, as only 31% of our cohort received a non-taxane regimen and 10% received a non-anthracycline regimen). Five-year RFS was 27% for patients with *BRCA1* PM versus 62% for patients without *BRCA*1 PM, (p=0.041, log rank test). Five-year OS was 36% for patients with *BRCA1* PM versus 77% for patients without *BRCA1* PM, (p=0.004, log rank test). The Kaplan-Meier plots for RFS and OS by methylation status are shown in Figure 5. RFS and OS remained significant after excluding the four patients (three *BRCA1*-unmethylated, one *BRCA1*-methylated) who did not receive an anthracycline as part of systemic therapy (data not shown).

Table 3 summarizes the results of the multivariable Cox proportional hazards models for RFS and OS. Included in the models were variables identified as significant (p<0.05) by univariate analysis (i.e., African-American race, stage 3 disease, node positivity, lymphovascular invasion, methylation status and *BRCA1* mRNA expression). In addition to African-American race and node positivity, presence of *BRCA1* PM was associated with a worse RFS (HR: 3.5, 95% CI: 1.3-9.8, p=0.016) and OS (HR: 6.2, 95% CI: 2.0-19.4, p=0.002) when compared to patients without *BRCA1* PM. *BRCA1* mRNA expression, stage and lymphovascular invasion were not significant predictors for RFS and OS in the multivariable model.

### *BRCA1* expression and outcome

**Figure 6:**
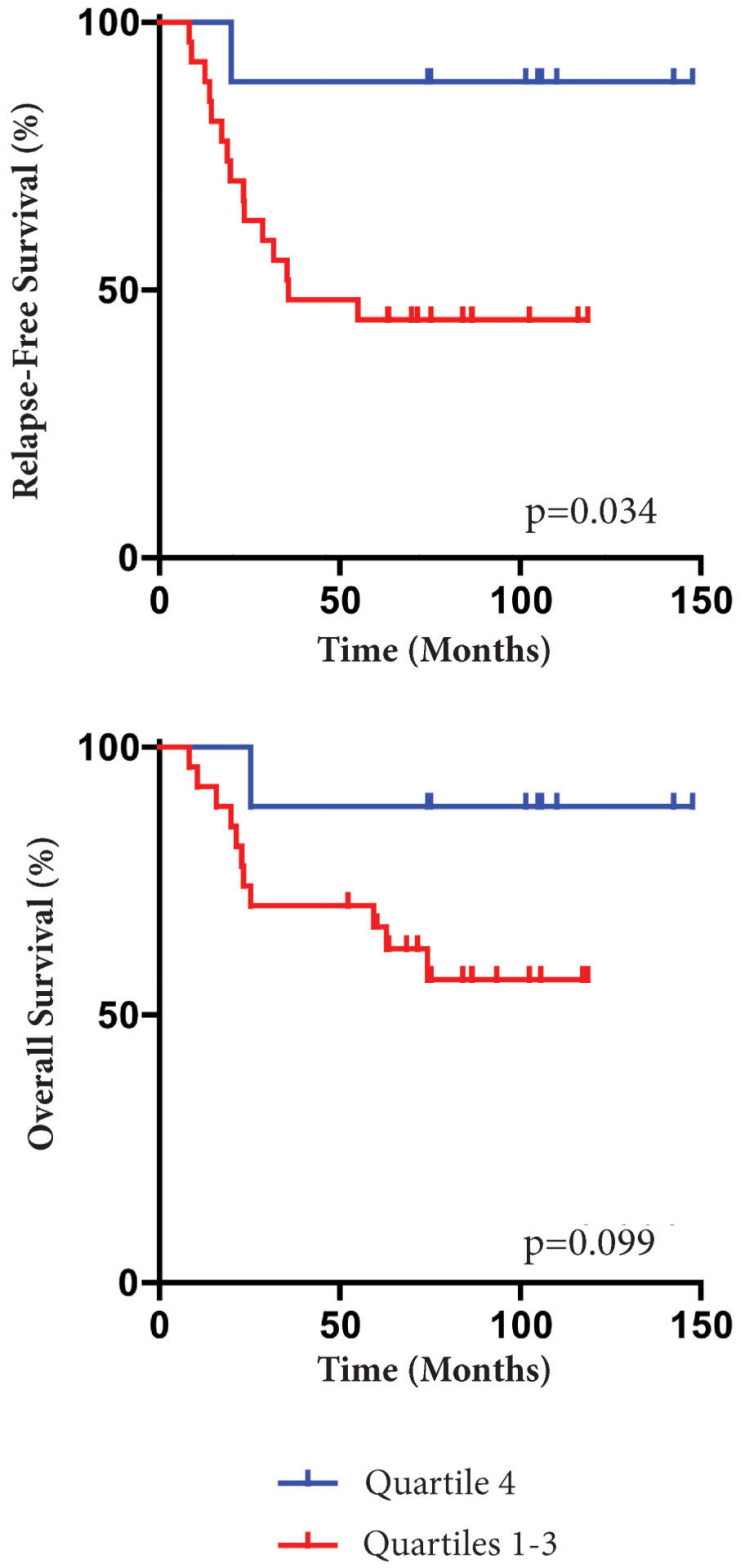
Kaplan-Meier survival plots for recurrence-free survival (RFS) and overall survival (OS) by *BRCA1* mRNA quartiles. Tick marks denote time of censoring.

We also examined the impact of *BRCA1* mRNA expression on RFS and OS. Five-year RFS was 44% for patients with *BRCA1* expression in the lowest three quartiles compared to 89% for patients with *BRCA1* expression in the highest quartile (p=0.034, log rank test). While the same trend was maintained for OS (Five-year OS 67% lowest three quartiles; 89% highest quartile), the trend was not statistically significant (p=0.099, log rank test). The Kaplan–Meier plot for RFS by *BRCA1* mRNA quartiles is shown in Figure 6. *BRCA1* mRNA expression was not a significant predictor of RFS and OS in the multivariable model. Thus, *BRCA1* PM was a more robust prognostic indicator compared to *BRCA1* expression in our data set.

At present, the TCGA breast cancer dataset has a short median follow-up (17 months) and a small number of overall survival events, limiting the utility of this dataset in performing survival analyses [9].

## Discussion

It is well established that patients with TNBC have a worse outcome compared to patients with other breast cancer subtypes [1,5,6,26]. One of the challenges in developing newer agents for treatment of TNBC has been lack of predictors of resistance to standard chemotherapy, as routine clinical and pathological variables do not clearly identify TNBC patients who are likely to develop recurrence with standard therapy. In this unselected cohort of TNBC patients who were treated with modern chemotherapy regimens, we have demonstrated that *BRCA1* PM can be used as a marker to identify patients who are destined to have a poor outcome. *BRCA1* PM was detected in 30% of subjects with TNBC, was associated with lower *BRCA1* transcript levels (suggesting epigenetic silencing* of BRCA1* gene) and was an independent predictor of poor outcome. Low *BRCA1* expression, although associated with inferior RFS in univariate analysis, was not an independent predictor in the multivariable model. We believe that the small size limited our ability to adequately evaluate a continuous marker such as *BRCA1* expression.

Our study adds to the existing data on the prognostic impact of *BRCA1* PM and expression in patients with TNBC. Although small, this is the first study to evaluate the prognostic impact of *BRCA1* PM in context of modern chemotherapy (70% of our cohort received both Anthracycline and taxane). In a recent publication, Xu et al., evaluated the impact of *BRCA1* PM on outcome in Chinese breast cancer patients [20]. *BRCA1* PM was detected in 30% of TNBC patients, and in a subgroup of chemotherapy treated patients *BRCA1* PM was associated with poor outcome in non-TNBC patients and better outcome in TNBC patients. These findings are in contrast to our study. Methylation of *BRCA1* promoter leads to *BRCA1* gene silencing and there is no preclinical data to suggest that the biological therapeutic sequelae of *BRCA1* silencing depends on the subtype of breast cancer. Thus, the reasons underlying the differential impact of *BRCA1* PM on chemotherapy response in triple negative and non-triple negative breast cancer in Xu et al., study are not clear. Furthermore, *BRCA1* mRNA expression analysis to confirm gene silencing is not reported Xu et al., cohort. Differences in chemotherapy regimens between the two cohorts can also explain the disparity between the findings. The majority of patients in the Xu et al., study were treated in 1990s and received non-anthracycline/taxane based chemotherapy, whereas most of our patients received anthracycline/taxane based therapy.

Although important, our study has several limitations. This is a small, retrospective study and results are subject to bias due to the retrospective nature and small sample size [27]. Our findings need to be confirmed in other larger independent cohorts. We do not have germline *BRCA1* information on all patients. Only 33% of the cohort underwent commercial *BRCA* germline testing, and none were found to carry a *BRCA* mutation. It is possible that the presence of unidentified *BRCA* germline mutations in untested patients impacted our results. It has previously been shown that the presence of a *BRCA1* germline mutation and *BRCA1* PM typically do not co-exist in the same tumor (i.e., *BRCA1* PM is not observed in *BRCA1* germline mutation associated tumors) [9,11,28,29]. Thus, co-existence of germline *BRCA* mutations are unlikely to contribute to the poorer outcome observed in our patients with methylated tumors. In the majority of prior studies, the outcomes of *BRCA* mutation-associated breast cancers are reported to be similar to patients with sporadic breast cancer [30-34]. Thus, it is also unlikely that the superior outcome observed in patients with unmethylated tumors in our study was driven by presence of *BRCA* germline mutations in this group.

While the present study does not mechanistically explain the poorer outcome in *BRCA1*-methylated tumors, several potential explanations can be evoked. We were the first to show that decreased expression of *BRCA1* occurs in sporadic breast cancer at the transition from ductal carcinoma *in situ* to invasive ductal carcinoma, and that experimental knockdown of *BRCA1* expression leads to accelerated growth of both normal and malignant mammary epithelial cells [35]. Several recent studies have suggested that loss of *BRCA1* expression or function leads to expansion of cell populations with stem/progenitor-like properties which classically are resistant to chemo-radiotherapy [36,37]. Lastly, and by direct extension, it is now appreciated that loss of *BRCA1*, and indeed the acquisition of a stem/progenitor-like phenotype in general, is associated with the migratory and invasive characteristics of the epithelial-to-mesenchymal transition (EMT) [38,39]. Thus, epigenetic inactivation of *BRCA1* in sporadic TNBC may manifest an intrinsically more aggressive and invasive tumor phenotype through multiple mechanisms, including increased growth, expansion of stem/progenitor-cells, activation of pro-invasive gene expression programs, and reduced therapeutic sensitivity to standard chemotherapy.

It is also possible that anthracycline- and taxane-based chemotherapy are not the ideal drugs to therapeutically capitalize on *BRCA1* insufficiency brought about by epigenetic *BRCA1* silencing. Though anthracycline agents induce double-strand breaks, repair of these lesions appears to require non-homologous end joining, an error-prone double strand break repair pathway that does not require *BRCA1*, and preclinical data suggests that anthracyclines do not exhibit selective toxicity in *BRCA1*-deficient cells [40-42]. Conversely, repair of platinum-induced interstrand crosslinks invokes *BRCA1*-mediated homologous recombination, and there is abundant clinical and *in vitro* evidence that *BRCA1*-deficient cells are hypersensitive to platinum agents [41-44]. Taxanes are an integral part of chemotherapy regimens for breast cancer treatment and appear to contribute particularly among patients with early stage TNBC [45,46]. However, the relative efficacy of taxanes in TNBC may be impacted by *BRCA1* functional state. In response to the abnormal mitosis induced by taxanes, *BRCA1* induces the mitotic spindle checkpoint and triggers apoptosis [47]. In the absence of functional *BRCA1*, this checkpoint is not activated and cells proceed through mitosis. Several *in vitro* studies suggest that *BRCA1*-deficient cells are resistant to microtubule poisons [47,48]. Supporting these pre-clinical findings, a recent retrospective analysis demonstrated that *BRCA1* mutation-associated advanced TNBCs are less sensitive to single-agent taxanes than sporadic TNBCs [49,50]. These data imply that anthracycline/taxane combination therapy may not be optimal for patients with *BRCA1*-deficient tumors. We speculate that the poorer outcome we observed in *BRCA1*-methylated tumors resulted from the aggressive biological features imbued by *BRCA1* deficiency in the context of chemotherapy that does not exploit the homologous recombination defect present in *BRCA1*-deficient cells.

While TNBC patients with *BRCA1*-methylated tumors may be destined to have poor outcome with standard anthracycline/taxane-based chemotherapy, genetic or epigenetic loss of *BRCA1* may be an Achilles’ heel that can be harnessed for therapeutic advantage. It is now appreciated in the ovarian cancer literature that upon treatment with standard platinum-based chemotherapy, *BRCA1*- and *BRCA2*-associated malignancies have an improved prognosis compared to sporadic epithelial ovarian cancers [44]. Thus, use of platinum salts may be a more rational treatment approach in breast cancers with either genetic or epigenetic inactivation *BRCA1*. Indeed, platinum compounds are more effective than anthracyclines in treating tumors arising in a *Brca1/p53* mouse model of spontaneous breast cancer [41,51]. Furthermore, recent *in vitro* and animal data suggests that epigenetic inactivation of *BRCA1* leads to the same degree of sensitivity to platinum agents as observed in presence of *BRCA1* mutations [52]. In recent years, poly(ADP-ribose) polymerase (PARP) inhibitors have been enthusiastically evaluated for treatment of *BRCA* mutation-associated and sporadic TNBCs. Although clinical trials of PARP inhibitors have demonstrated encouraging activity in *BRCA* mutation-associated breast cancers, to date PARP inhibitors have failed to demonstrate significant activity in unselected patients with sporadic TNBC [51]. Thus, there is a need to define markers that can identify sporadic TNBC tumors which are likely to benefit from this novel class of drugs. It might be possible to extend the observation of PARP inhibitor sensitivity of *BRCA1*/*BRCA2* mutation-associated tumors to sporadic *BRCA1*-hypermethylated tumors. Indeed, *in vitro* data suggests that *BRCA1* hypermethylation confers the same degree of sensitivity to PARP inhibitors as does *BRCA1* mutation [14].

## Conclusion

Our study shows that *BRCA1* PM occurs frequently in TNBC and that epigenetic *BRCA1* silencing is associated with poor outcome in presence of modern anthracycline/taxane-based chemotherapeutic regimens. Whether *BRCA1* PM is indeed a robust prognostic biomarker in this regard needs to be confirmed in larger cohorts from prospective clinical trials. If validated in larger cohorts, *BRCA1* PM can serve as a clinically useful biomarker to identify TNBC patients who are likely to experience suboptimal outcomes with standard chemotherapy. *BRCA1* PM may potentially also be used as a patient selection criterion for to identify patients who may benefit from therapeutic approaches which target *BRCA1* deficiency, including platinum compounds and/or PARP inhibitors.

## Figures and Tables

**Table 1:** Baseline characteristics

Characteristics	Total	*BRCA1* methylation*		P-value
		Methylated	Unmethylated	
N	39	11	26	--
Median age at diagnosis (range)	52 (33-80)	47 (33-61)	53 (39-80)	0.067

**Ethnicity**				0.40
Caucasian	29 (74%)	8 (73%)	19 (73%)	
African American	7 (18%)	3 (27%)	4 (15%)	
Hispanics	3 (8%)	0	3 (12%)	

**Lymph Node status**				1.00
Negative	20 (51%)	5 (46%)	13 (50%)	
Positive	19 (49%)	6 (54%)	13 (50%)	

**Pathological TNM Stage**				0.47
I	12 (30%)	2 (18%)	9 (35%)	
II	15 (39%)	4 (36%)	10 (39%)	
III	12 (31%)	5 (46%)	7 (27%)	

**Lymphovascular invasion**				0.72
Present	18 (47%)	5 (46%)	12 (46%)	
Absent	20 (53%)	6 (54%)	14 (54%)	

**Histology**				0.30
Ductal	38 (97%)	10 (91%)	26 (100%)	
Other	1 (3%)	1 (9%)	0 (0%)	

**Surgery type**				1.00
Breast conservation	19 (49%)	5 (46%)	12 (46%)	
Mastectomy	20 (51%)	6 (54%)	14 (54%)	

**Chemotherapy**	39(100%)	11(100%)	26(100%)	--
Adjuvant	30(74%)	6(54%)	22(85%)	0.09
Neoadjuvant	9(26%)	5(46%)	4(15%)	--
Type of Chemotherapy	100%	100%	100%	--
Anthracycline	35 (90%)	10(91%)	23(89%)	1.00
Taxane	27 (69%)	8(73%)	18 (69%)	1.00

* *BRCA1* PM MSPCR was successful in 37/39 specimens

**Table 2:** Recurrence Free Survival and Overall Survival estimates

Recurrence Free Survival					

	*n* at risk	*n* events	5-year RFS	95% CI	*p* value*
All	39	18	52%	(38%, 70%)	--

**Race**					

AA	7	6	14%	(0%, 40%)	0.015t
Not AA	32	12	62%	(46%, 79%)	--
White	29	11	62%	(45%, 80%)	0.051
Other	3	1	67%	(13%, 100%)	--

**Age** ‡					

Below median	19	11	42%	(20%, 64%)	--
Above median	20	7	65%	(44%, 86%)	0.22

**Pathological TNM Stage**					

I and II	27	8	70%	(53%, 88%)	--
III	12	10	16%	(0%, 38%)	<0.001

**Lymphovascular invasion**					

Present	18	11	39%	(16%, 61%)	0.050
Absent	20	6	70%	(50%, 90%)	--

**Lymph Node status**					

Node Negative	20	4	80%	(63%, 97%)	--
Node Positive	19	14	26%	(7%, 46%)	<0.001

**BRCA1 PM Status**					

Not methylated	26	10	61%	(43%, 80%)	--
Methylated	11	8	27%	(1%, 54%)	0.041

**BRCA1 expression** §					

Lower three quartiles	27	15	44%	(27%, 62%)	--
Highest Quartile	9	1	89%	(72%, 100%)	0.34

Overall Survival					

	*n* at risk	*n* events	5-year OS	95% CI	*p* value*
All	39	14	69%	(55%, 84%)	--

**Race**					

AA	7	5	27%	(0%, 62%)	0.011f
Not AA	32	9	78%	(64%, 92%)	--
White	29	8	79%	(65%, 94%)	0.036
Other	3	1	67%	(13%, 100%)	--

**Age** ‡					

Below median	19	9	57%	(35%, 80%)	--
Above median	20	5	80%	(63%, 97%)	0.19

**Lymph Node status**					

Negative	20	2	90%	(76%, 100%)	--
Positive	19	12	47%	(25%, 70%)	<0.001

**Pathological TNM Stage**					

I and II	27	5	90%	(77%, 100%)	--
III	12	9	25%	(1%, 50%)	<0.001

**Lymphovascular invasion**					

Present	18	9	61%	(39%, 84%)	0.12
Absent	20	5	75%	(56%, 94%)	--

**BRCA1 PM Status** □					

Not methylated	26	6	81%	(66%, 96%)	--
Methylated	11	8	36%	(8%, 65%)	0.004

**BRCA1 expression** §					

Lower three quartiles	27	11	67%	(49%, 84%)	--
Highest Quartile	9	1	89%	(72%, 100%)	0.99

* Log rank test;

† AA compared to non AA;

‡ Median age 52 years;

§ BRCA1 PM MSPCR assay was successful in 37/39 subjects;

□ BRCA1 mRNA qRT-PCR was successful in 36/39 subjects

**Table 3:** Multivariable Cox proportional hazards models*

RFS estimates			

	HR	95% CI	P
**BRCA1 PM Status**			

Not methylated	1.0	(1.3, 9.8)	0.016
Methylated	3.5	--	--

**Race**			

Non AA	1.0	(1.6, 12.9)	0.006
AA	4.5	--	--

**Nodal Status**			

Negative	1.0	(2.7, 31.8)	<0.001
Positive	9.3	--	--

OS estimates			

	HR	95% CI	P
**BRCA1 PM Status**			

Not methylated	1.0	(2.0, 19.4)	0.002
Methylated	6.2	--	--

**Race**			

Non AA	1.0	(2.2, 31.1)	0.002
AA	8.3	--	--

**Nodal Status**			

Negative	1.0	93.2-86.10	0.001
Positive	16.7	--	--

* Variables included: race, pathological stage, node positivity, lymphovascular invasion *BRCA1* expression and methylation status. Stage, lymphovascular invasion and *BRCA1* expression were not significant in the multivariable model.
